# Ductility Enhancement of Post-Northridge Connections by Multilongitudinal Voids in the Beam Web

**DOI:** 10.1155/2013/515936

**Published:** 2013-11-07

**Authors:** Sepanta Naimi, Murude Celikag, Amir A. Hedayat

**Affiliations:** ^1^Department of Civil Engineering, Istanbul Aydin University, Florya Main Campus, İnönü Street, Sefaköy-Küçükçekmece, 34295 Istanbul, Turkey; ^2^Department of Civil Engineering, Eastern Mediterranean University, P.O. Box 95, Gazimagusa, North Cyprus, via Mersin 10, Turkey; ^3^Department of Civil Engineering, Islamic Azad University, Kerman Branch, Joopar Road, Kerman 7635131167, Iran

## Abstract

Since the earthquakes in Northridge and Kobe in 1994 and 1995, respectively, many investigations have been carried out towards improving the strength and ductility of steel beam to column pre- and post-Northridge connections. In order to achieve these objectives, recent researches are mainly focused on three principles: reducing the beam section to improve the beam ductility, adding different kinds of slit damper to beam and column flanges to absorb and dissipate the input earthquake energy in the connection and strengthening the connection area using additional elements such as rib plates, cover plates, and flange plates to keep the plastic hinges away from the column face. This paper presents a reduced beam section approach via the introduction of multilongitudinal voids (MLV) in the beam web for various beam depths varying from 450 mm to 912 mm. ANSYS finite element program was used to simulate the three different sizes of SAC sections: SAC3, SAC5, and SAC7. Results showed an improvement in the connection ductility since the input energy was dissipated uniformly along the beam length and the total rotation of the connection was over four percent radian.

## 1. Introduction

**Figure 1:**
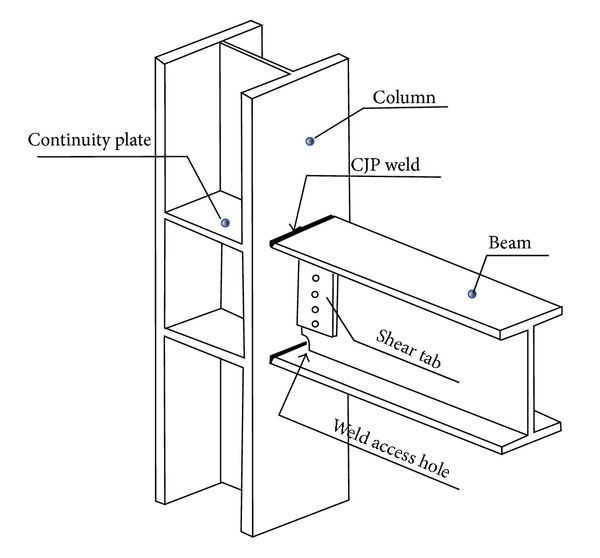
Typical pre-Northridge beam-to-column moment connection.

Steel moment resisting frames had extensive brittle fractures in their welded connections during the earthquakes in Northridge (1994) and Kobe (1995) [1]. Since then modifications to design procedure of pre-Northridge connections and its welding has been introduced. E70T-4 type welding has been changed to E70-TGK2 with smooth welding access holes and backing bar removed from the bottom beam flange [2, 3]. This type of connection is now known as post-Northridge connection. The typical pre-Northridge connection is shown in Figure 1.

Three principles are mainly used to improve the strength and ductility of the post-Northridge connections

- Strengthening of connection by adding additional elements including cover plates and flange plates [4, 5], triangular haunches [6], straight haunches [7], upstanding ribs [8], lengthened ribs [9], side plates [10], and bolted brackets [11, 12].
- Reducing the beam section to improve the beam ductility so that the stress concentration will transfer to a region away from the connection. Reduction of beam section can be done by reducing the flange section (reduce beam section, RBS [13]) or by reducing the web section (reduce beam web, RBW). RBW connections include the wedge design beam connections [14, 15] and reduced beam web with circular voids [16, 17], rectangular long voids [18], drilled voids [19], and RBW with arch-shape cuts at the beam web [20].
- Adding different kinds of slit damper plates to beam and column flanges that will absorb and dissipate energy at connections during earthquake [21].

These methods are applied to shift the plastic hinge from the connection area at the face of the column to the beam so that the stress concentration will be reduced at the complete joint penetration area (CJP). These modifications must be as such to be applicable for both existing and new buildings. Weakening of the beam section (RBS) at the flange area in existing buildings is difficult and is expected to be more costly than reducing the beam web (RBW). This is due to difficulties in accessing the beam top flange and modifying it in the presence of concrete floor. 

**Figure 2:**
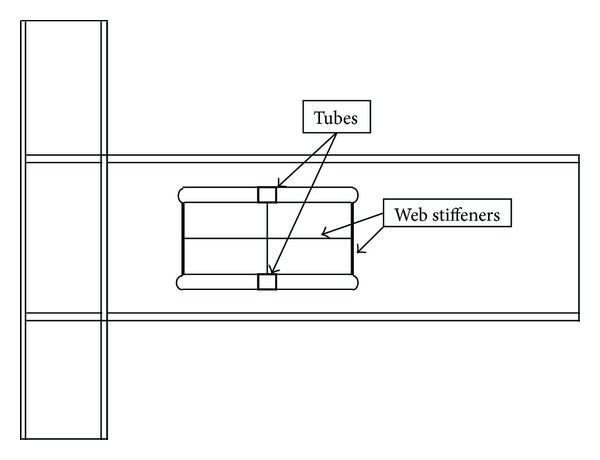
Single longitudinal voids with stiffeners and tubes at the center of voids proposed by Hedayat and Celikag [18].

In 2009, Hedayat and Celikag [18] proposed the use of rectangular long voids at the beam web to enhance the connection ductility of post-Northridge connections (Figure 2). This method was effective for beams with the maximum depth equal to 600 mm. However, for deeper beams due to the high level of strain concentration at the RBW area and excessive lateral-torsional buckling of the beam web (which was due to the increase in the depth of the voids), the efficiency of this method reduced and the modified connection did not achieve adequate connection's strength and ductility. Hence, for deep beams, Hedayat and Celikag [18] proposed to add tube and stiffener at the RBW area. However, the main drawback of this approach is the increase in cost and time consumption to modify the beam.

**Figure 3:**
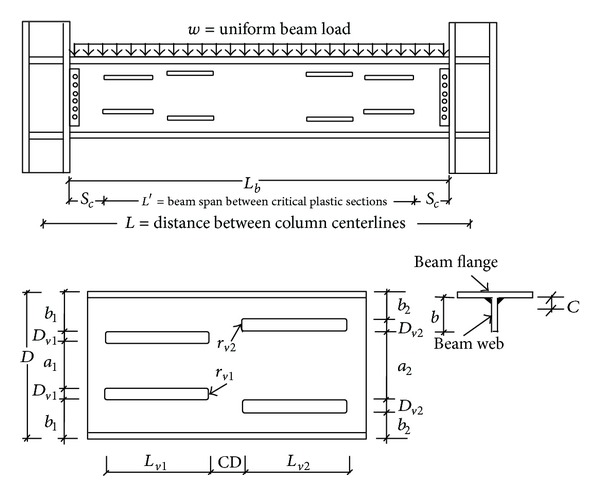
Modified post-Northridge connections with multilongitudinal voids.

This study was aimed at increasing the strength and ductility of post-Northridge connections with deep beams by creating multilongitudinal voids at the beam web (Figure 3). When compared to the method presented in [18], this method is more economical with less cost and workmanship. This method also can lead to the achievement of a more uniformly distributed strain at the RBW area when compared to the one proposed in [18]. To find out the best connection configuration, a parametric study was done with respect to the size and the location of the voids. This parametric study was carried out through the 144 models and it was based on the finite element method.

## 2. Finite Element Method

Three nonmodified post-Northridge connections, comprised of three pretested specimens SAC3 (beam: W24 × 68; column: W14 × 120), SAC5 (beam: W30 × 99; column: W14 × 176), and SAC7 (beam: W36 × 150; column: W14 × 257) from Lee et al. [3] were modeled using the general purpose finite element program ANSYS [22]. These specimen sizes were chosen since they are considered as good representatives of the conventional pre-/post-Northridge specimen sizes, small, medium and large size connections [3]. The length of the beam (*L*_*b*_/2) and the column for all these specimens were 3429 mm and 3658 mm, respectively. Modulus of elasticity and Poisson's ratio are taken as 200 kN/mm^2^ and 0.3, respectively. Other geometric parameters and all the other material properties of these specimens are summarized in Tables 1 and 2, respectively. The proposed beam end configuration with different values of design parameters was then applied to all these nonmodified post-Northridge connections to create modified specimens. 

After Northridge earthquake, Miller [2] inspected more than 100 damaged buildings, and also experimental tests were conducted by the SAC group (e.g., [3]) on the pre- and the post-Northridge connections; all there showed that, the failure of this type of connection is not often due to the failure of bolts. Therefore, in the finite element model, the bolts were not exactly modeled, but shear tab, bolt holes, and interaction between the shear tab and the beam web were modeled to achieve a realistic model. In finite element models, both welds and base metals were modeled using shell elements, and the associated material property was defined for each one. SHELL43 was used to model weld, shear tab, continuity plates and column plates, whereas SHELL181 was used to model the beam plates. SHELL43 and SHELL181 are one-layer four-node and multi-layer eight-node shell elements, respectively. These elements have six degrees of freedom at each node and all of them have plasticity, large deflection, and large strain capabilities. In the case of using SHELL181, each element was separated into five layers across the thickness. The number of layers was selected based on the finite element study carried out by Gilton and Uang [23]. 

**Figure 4:**
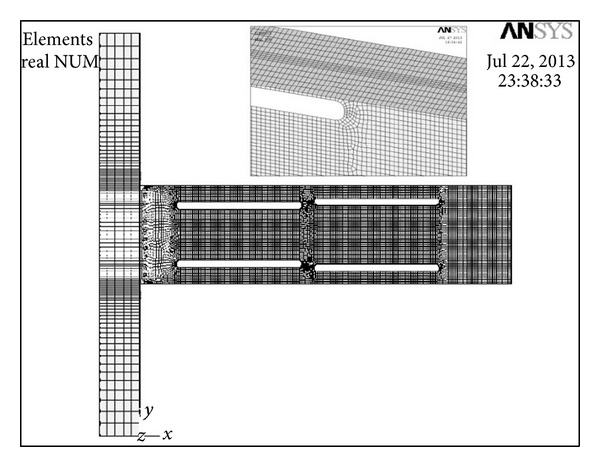
Typical finite element mesh of a RBW with multilongitudinal voids.

In order to determine the appropriate mesh density, a mesh sensitivity study was done for both modified and nonmodified specimens based on the recommendation given by the ANSYS program and by comparing the analytical results with experimental results of [3].  Figure 4 shows the finite element mesh for a typical modified specimen with multilongitudinal voids. A very fine mesh size was used for the beam flange and web at the voids area to accurately capture the local buckling of the beam flange and web at this region. The number of elements for specimens (SAC3, SAC5, and SAC7) in average was 27000. Around 30% to 50% of this amount was due to the size of the voids located at the beam web. 

**Figure 5:**
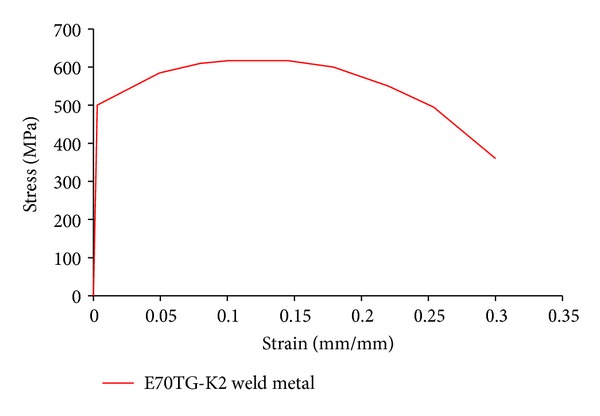
Stress-strain relationship used for the weld metal (Mao et al. [24] and Ricles et al. [25]).

To perform material nonlinearity analyses, plasticity behavior was based on the Von-Mises yielding criteria and the associated flow rule. Isotropic hardening was assumed for the monotonic analysis, whereas kinematic hardening was assumed for the cyclic analysis as used by Mao et al. [24] and Ricles et al. [25]. A bilinear material response with a postyielding stiffness equal to 4% of the modulus of elasticity of steel was used for the base metals in accordance with the material properties given by Lee et al. [3]. For weld metals, a multilinear material response (Figure 5) based on the material property given by Mao et al. [24] and Ricles et al. [25] was used. The analyses with monotonic loading were conducted by applying a monotonic vertical displacement load to the beam tip until achieving more than 4% total rotation at the column web center, whereas the load history recommended by FEMA [26] was utilized for analyses with cyclic loading. When applied loads are in the vertical direction only, then the out-of-plane deformations (normal to the web) may not occur. Therefore, in order to ensure that buckling occurs when the model becomes unstable, the imperfect model was analysed under cyclic or monotonic loadings. In this study, in order to determine the imperfect model, first the buckling mode shapes were computed in a separate buckling analysis and then were implemented to perturb the original perfect geometry of the model as it was done by SAC group [5]. 

In order to verify the validity of the numerical research, Hedayat and Celikag [17, 18] prepared finite element models for the specimens SAC3, SAC5, and SAC7 of the experimental study conducted by Lee et al. [3]. The numerical results agreed suitably with the experimental ones.

## 3. The Proposed Beam End Configuration (BEC)

### 3.1. Details of the Proposed Beam End Configuration (BEC)

Details of the proposed BEC are shown in Figure 3. Effective parameters in the geometry of the proposed BEC are listed as follows:

- *D*_*v*1_: depth of the first pair of voids,
- *D*_*v*2_: depth of the second pair of voids,
- *L*_*v*_: length of each void,
- *a*_1_: perpendicular clear distance between the first pair of voids,
- *a*_2_: perpendicular clear distance between the second pair of voids,
- *D*: beam overall depth, and also the horizontal distance from the face of the column to the center of the first pair of voids.

The equation for minimum required shear depth (see (1)) [27] can be used to determine the minimum clear vertical distance, parameters *a*_1_ and *a*_2_, between the two voids. Consider

(1) $$\begin{array}{c} \phi R_{n}=0.9\times 0.6\times f_{y}\times A_{g}, \end{array}$$

where *ϕR*_*n*_ is total shear force which is equal to the expected beam plastic moment capacity at column face divided by half of the total beam length (*L*_*b*_/2), *f*_*y*_ is the beam nominal yield strength, and *A*_*g*_ is gross shear area (*A*_*g*_ = *a* × *t*_*w*_, *t*_*w*_ is the beam web thickness). If the over strength factor is taken as 1.2 [28], then (1) can be simplified to find the minimum required shear depth, *a*_1_ and *a*_2_, as follows:

(2) $$\begin{array}{c} a_{1}=\frac{5.29\times Z_{b}}{L_{b}\times t_{w}}, \end{array}$$

where *Z*_*b*_ is the plastic section modulus of the beam. Parameter *a*_1_ is equal to 465 mm, 300 mm, and 210 mm for specimens SAC7, SAC5, and SAC3, respectively. The horizontal length of each void is 1.25 times the beam overall depth (*L*_*v*1_ = *L*_*v*2_ = 1.25*D*). The minimum value of parameter *b* (see Figure 3) is 1.4 times the parameter *c*, where *c* is the roots radius of the beam (*b* = 1.4*c*). The factors 1.25 and 1.4 were selected based on the parametric study done by Hedayat and Celikag [18] for RBW connections with single longitudinal voids. The first pair of voids was located as such that their distance from center of voids to the face of column was equal to the overall depth of the beam (Figure 3). Void depth *D*_*v*1_ was achieved by (3), where *t*_*f*_ is the beam flange thickness and *b*_1_ is as shown as the inset figure in Figure 3. In this study, for all cases, the voids of the same size were used (*D*_*v*1_ = *D*_*v*2_). Consider

(3) $$\begin{array}{c} D_{v1}=\left[\frac{\left(D-2t_{f}-a_{1}\right)}{\left[2\left(\left(b_{1}/D_{v1}\right)+1\right)\right]}\right]. \end{array}$$

The radii of the corners of rectangular voids (parameter *r*_*v*_) was obtained from [29] and (4). It were mentioned in [27] that *r*_*v*_ ≥ 2*t*_*w*_, where smallest *t*_*w*_ is 8 mm. However, in the same reference, *r*_*v*_ = 9.5 mm was permitted. On the other hand, to use small *r*_*v*_, smoother surface should be provided by drilling [29]. Consider

(4) $$\begin{array}{c} r_{v}\;\;\left(\text{mm}\right)=\frac{D_{v}-20\;\text{mm}}{2}. \end{array}$$

Other design parameters are defined in the next section. 

### 3.2. Design Parameters

All specimens SAC3, SAC5, and SAC7 were used for the parametric study which was carried out on the geometry of the voids by defining three design parameters, *α*, *β*, and *γ*. These parameters are defined as follows.

*Parameter* *α*. This parameter is ratio of *b*_1_ to *D*_*v*1_(*α* = *b*_1_/*D*_*v*1_ where *b*_1_ and *D*_*v*1_ are shown in Figure 3). The values used for parameter *α* were 2, 3, and 4. Hence, by assuming the value of this parameter the first pair of voids depth (parameter *D*_*v*1_) can be obtained by using (3). 

*Parameter* *β*. This parameter is the ratio of *b*_2_ to *b*_1_ which are shown in Figure 3 (*β* = *b*_2_/*b*_1_). Four different values were used for parameter *β*: 1, 0.75, 0.5, and 0.25. Hence, by assuming the value of this parameter and knowing *D*_*v*2_ (*D*_*v*2_ = *D*_*v*1_), the perpendicular distance of the second pair of voids (parameter *a*_2_) can be obtained. However, this value cannot be less than the value obtained from (2). It should be noted that, in this study, the value of parameter *a*_1_ was directly obtained from (2). 

*Parameter* *γ*. This parameter is the ratio of the horizontal clear distance between the first and the second voids to the void length (*γ* = *CD*/*L*_*v*_). The values used for parameter *γ* were 0.1, 0.15, 0.2, and 0.25.

## 4. Analytical Results

### 4.1. Typical Behavior of the Post-Northridge Connection in the Presence of the Proposed BEC

In order to enhance the strength and ductility of post-Northridge connections, Hedayat and Celikag [18] used one pair of longitudinal voids at the beam web (Figure 2). This modification was effective to limit the stress concentrations at the complete joint penetration (CJP) groove welds at the column face and to provide more energy dissipation along the beam length. Note that this method was effective for beams with the overall depth less than 750 mm. However, for deeper beams, where the depths of voids were large, this method alone was not adequate. Hence, for deep beams, in order to delay or prevent the beam web buckling and increase the connection ductility, they proposed the use of web stiffeners and tubes at the beam web area (Figure 2). 

**Figure 6:**
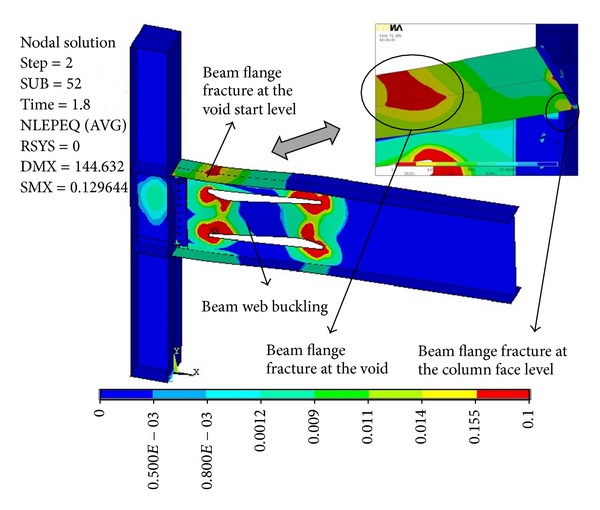
Plastic equivalent strain (PEEQ) distribution for modified specimen SAC7 with single voids at four percent total rotation.

Figure 6 shows the plastic equivalent strain (PEEQ) distribution for modified specimen SAC7 with deep beam W36 × 150 (beam overall depth = 912 mm) in the case of using a single pair of voids at four percent total rotation (sub step = 52 in ANSYS program). This figure clearly shows the PEEQ strain concentrations at the RBW area and the excessive lateral torsional buckling of the beam web which was due to the use of large voids at the beam web area. These finally led to the beam flange fracture at the void area before the achievement of four percent total rotation. 

**Figure 7:**
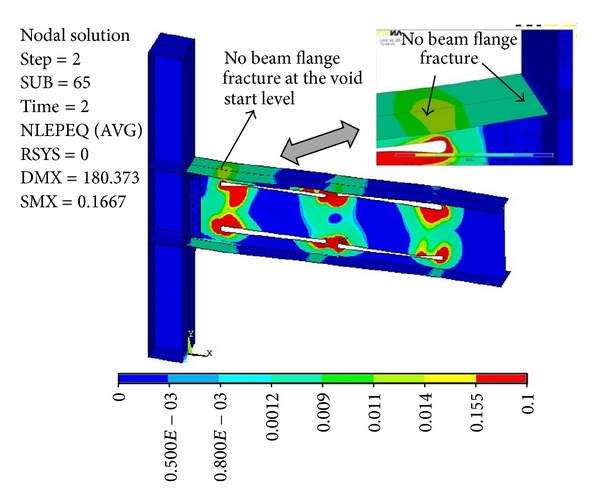
Plastic equivalent strain (PEEQ) distribution for modified specimen SAC7 with multivoids at five percent total rotation.

**Figure 8:**
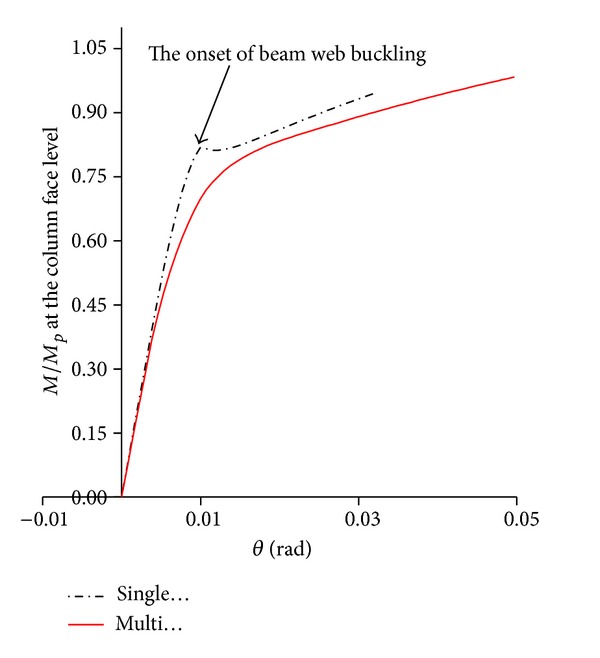
Normalize moment-rotation curves of modified specimens SAC7 with single and multilongitudinal voids.

Figure 7 shows the plastic equivalent strain distribution of the same specimen with multilongitudinal voids at five percent total rotation (sub step = 65 in ANSYS program). Note that, in this case, due to the use of multivoids, the depths of voids became smaller when compared to the ones used in Figure 6. As this figure shows, PEEQ strains are more uniformly distributed between multivoids, such that the normalized PEEQ (plastic equivalent strain divided by yield strain) at the most critical location of a connection (i.e., at the root of weld access hole) reduced from 92.77 for the beam with single pair of voids to 46.01 for multivoid specimen. In addition, multivoids caused a remarkable reduction in the plastic equivalent strain concentration at the beam flange at the start level of the first pair of voids (see Figure 7). It caused a remarkable delay in the connection failure time such that this specimen could easily achieve more than five percent total rotation at the column web center. The moment-rotation curve of this specimen is shown in Figure 8, where a remarkable delay is apparent in the onset of the beam web local buckling when compared to the same specimen with single voids. Figure 8 shows that the initial rotational stiffness of the two specimens is approximately the same. However, multivoids specimens have undergone earlier yielding in the yielding region, but they achieved much more ductility and strength when compared to the single pair of voids.

### 4.2. Effect of Design Parameters on the Strength and Ductility of the Modified Post-Northridge Connections

**Figure 9:**
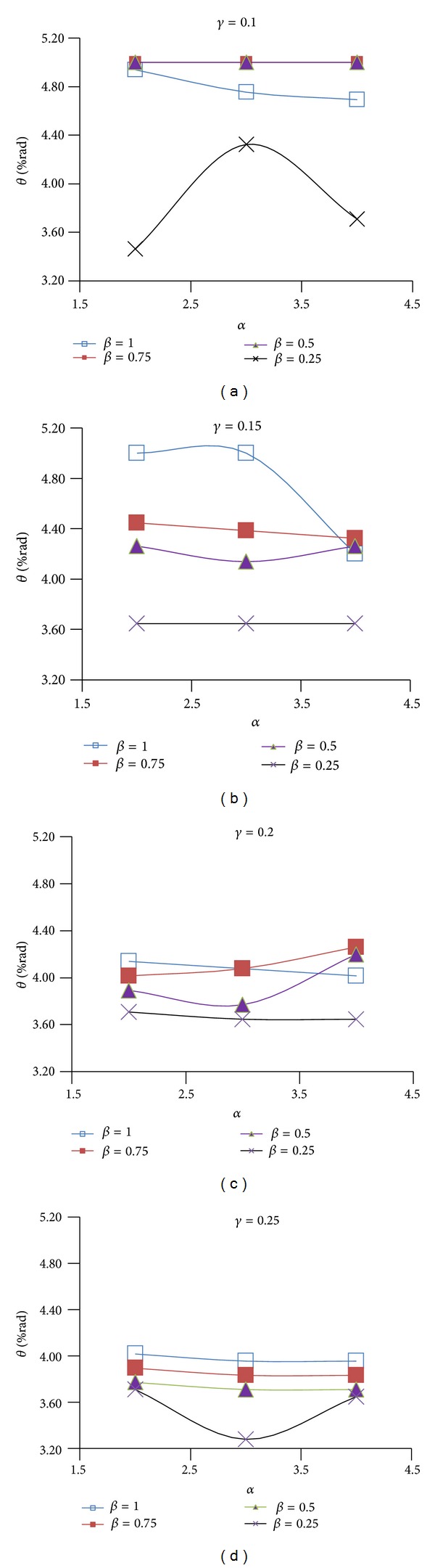
*θ* versus *α* for different values of *β* and *γ* for SAC7.

**Figure 10:**
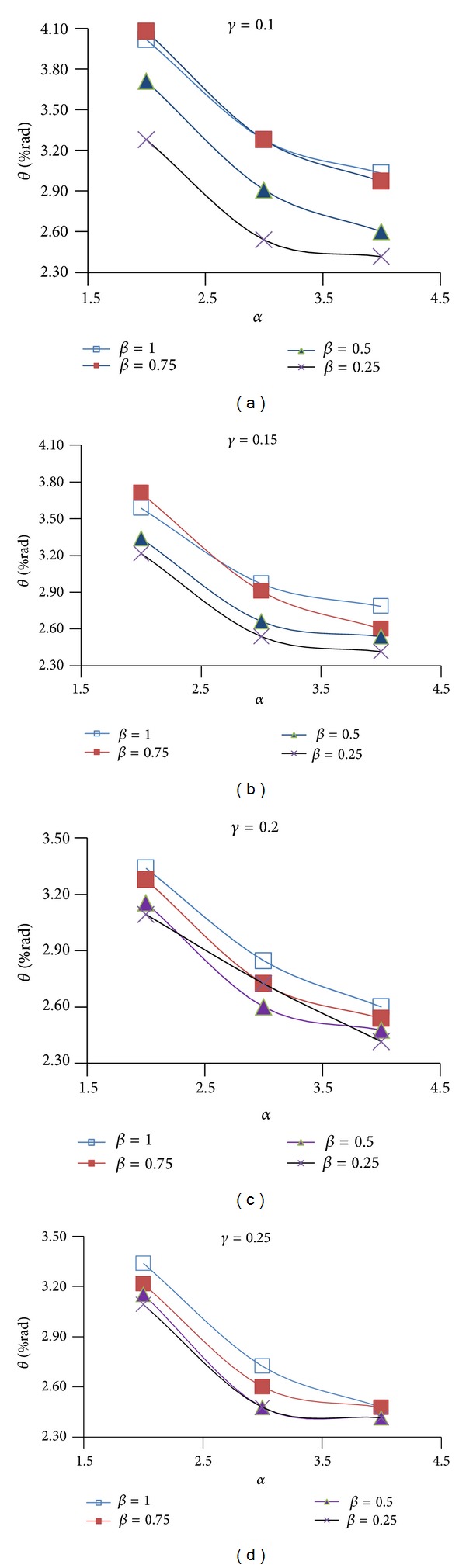
*θ* versus *α* for different values of *β* and *γ* for SAC5.

**Figure 11:**
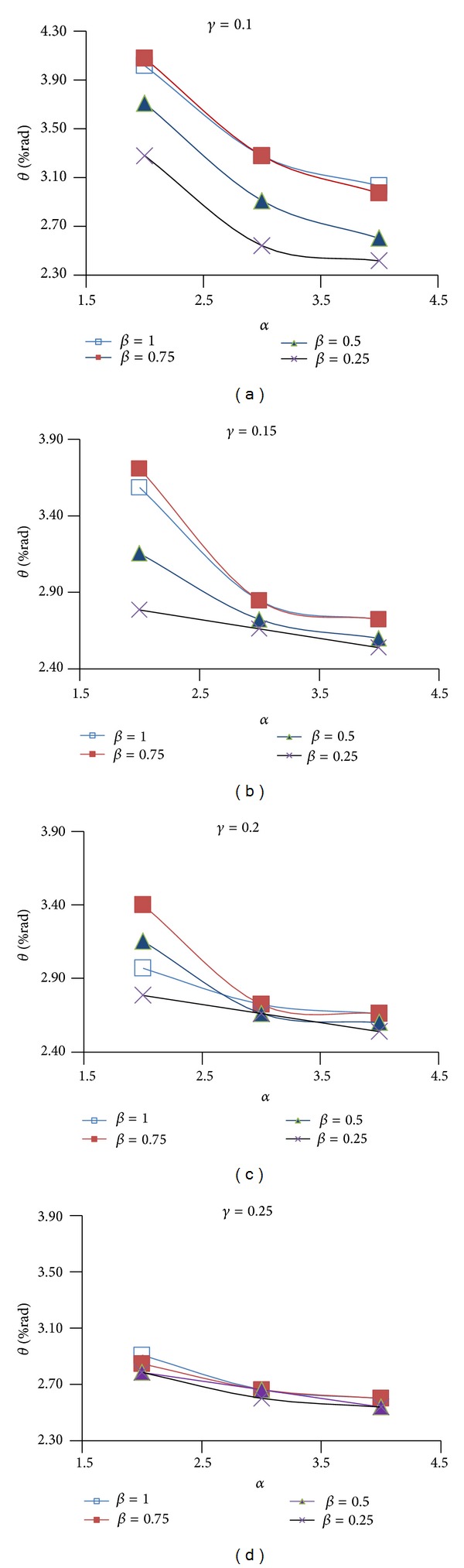
*θ* versus *α* for different values of *β* and *γ* for SAC3.

Figures 9, 10, and 11 show the effect of design parameters *α*, *β*, and *γ* on the ductility of the modified specimens SAC7, SAC5, and SAC3. Connection ductility was evaluated by using parameter *θ*_CWC_, which is the total rotation of the connection at the column web center. It is calculated by dividing the beam tip deflection by a distance measured from the beam tip to the column web center.

By increasing parameter *α*, the depth of the first pair of voids (i.e. parameter *D*_*v*1_) decreases and the depth of the T-sections (parameter *b*_1_) at the top and bottom of the rectangular voids increases. As Figures 9 through 11 shows for most of the specimens (except for few modified specimens of SAC7) by increasing this parameter, the connection ductility decreased which can be due to the reduction in the energy dissipation capacity of the beam at the first pair of voids area. Results indicate that the highest connection ductility was achieved when parameter *α* was equal to 2. It should be noted that the initial investigations showed that smaller value may not be desirable since it caused a remarkable reduction in the lateral-torsional/flexural stiffness of the T-sections at the top and bottom of the rectangular voids. It consequently promoted the onset of the torsional buckling of the beam web and flexural buckling of the T-sections. 

Initial investigations of the behavior of the proposed BEC showed that the second pair of voids must be located at a closer vertical distance to the beam flange surface when compared to the first pair of voids. This significantly helped to increase the efficiency of the second pair of voids to decrease the strain concentration at the region of the first pair of voids and consequently to uniformly distribute the plastic equivalent strains along the beam length. For this reason, the value of parameter *β* (*β* = *b*_2_/*b*_1_) varied between 1 and 0.25. By decreasing parameter *β* the value of parameter *b*_2_ decreased. Since the first and the second pair of voids have the same depth (*D*_*v*1_ = *D*_*v*2_), it caused an increase in parameter *a*_2_ and consequently moved the second pair of voids up. As it is clear from the Figures 9 to 11, the excessive decrease in parameter *β* was not desirable, since it caused a remarkable increase in the plastic equivalent strains at the beam flange at the second voids area, promoted the beam flange fracture at this area and finally caused a significant reduction in the connection ductility. The highest connection ductility was achieved for *β*, equal to 1 or 0.75.

By decreasing parameter *γ*, the horizontal distance between the voids reduces. This helped to increase the efficiency of the second pair of voids and reduced the strain concentrations at the column face region and at the area of the first pair of voids. However, excessive decrease in this parameter was also detrimental (i.e., *γ* less than 0.1) since it caused excessive beam web buckling in the area between the first and the second pair of voids and consequently reduced connection strength and ductility. Initial investigations indicated undesirable behavior of connection in the case of using parameter *γ* as less than 0.1. It should be noted that higher values of parameter *γ* were also undesirable since they significantly reduced the efficiency of the second pair of voids in uniformly distributing the plastic equivalent strains along the beam length and they caused an excessive increase in the strain concentration at the column face region and at the area of the first pair of voids. Based on this discussion, parameter *γ* varied between 0.1 and 0.25. Considering the finite element results in Figures 9 to 11 and the ductility of connection, it might be concluded that 0.1 is the optimum value for parameter *γ*.

**Figure 12:**
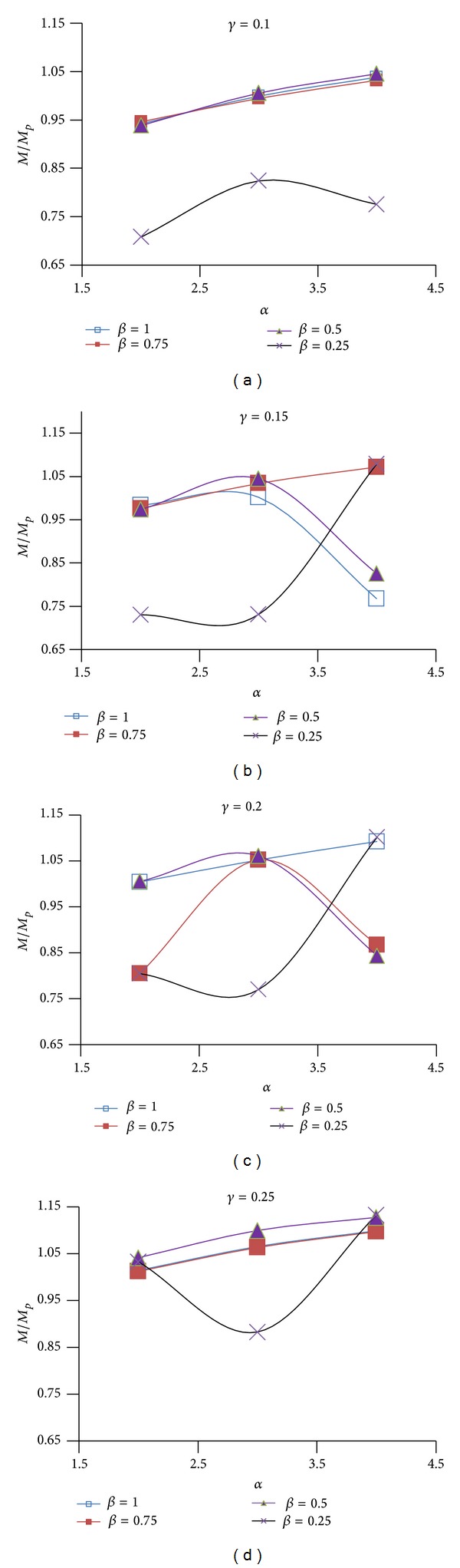
*M*/*M*_*P*_ versus *α* for different values of *β* and *γ* for SAC7.

**Figure 13:**
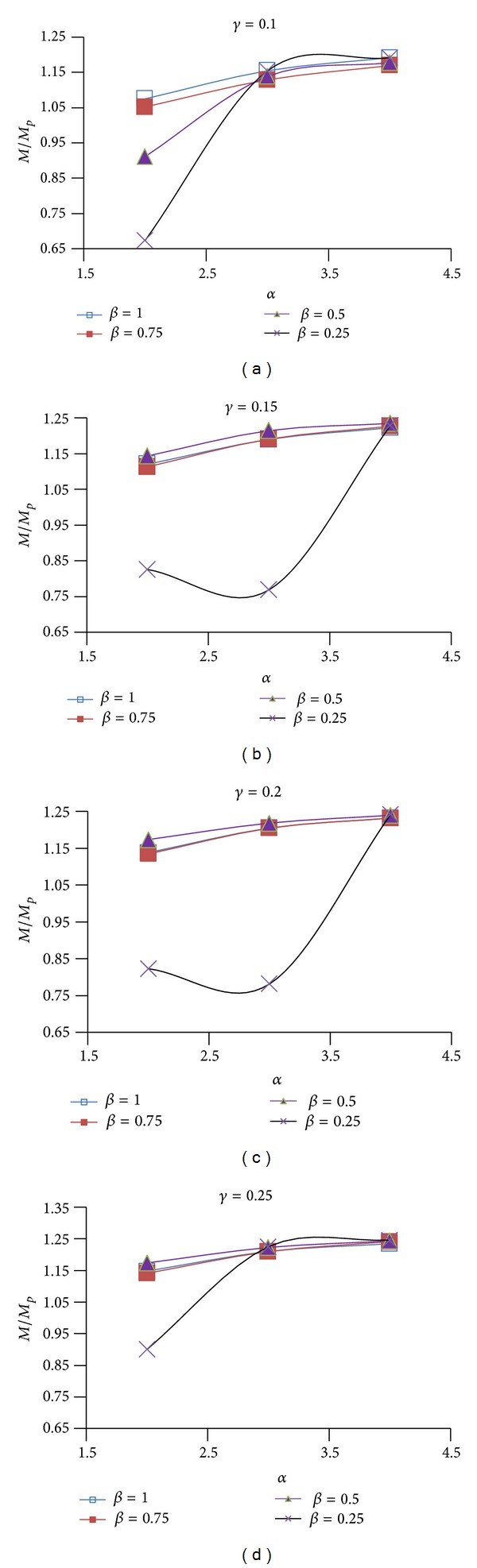
*M*/*M*_*P*_ versus *α* for different values of *β* and *γ* for SAC5.

**Figure 14:**
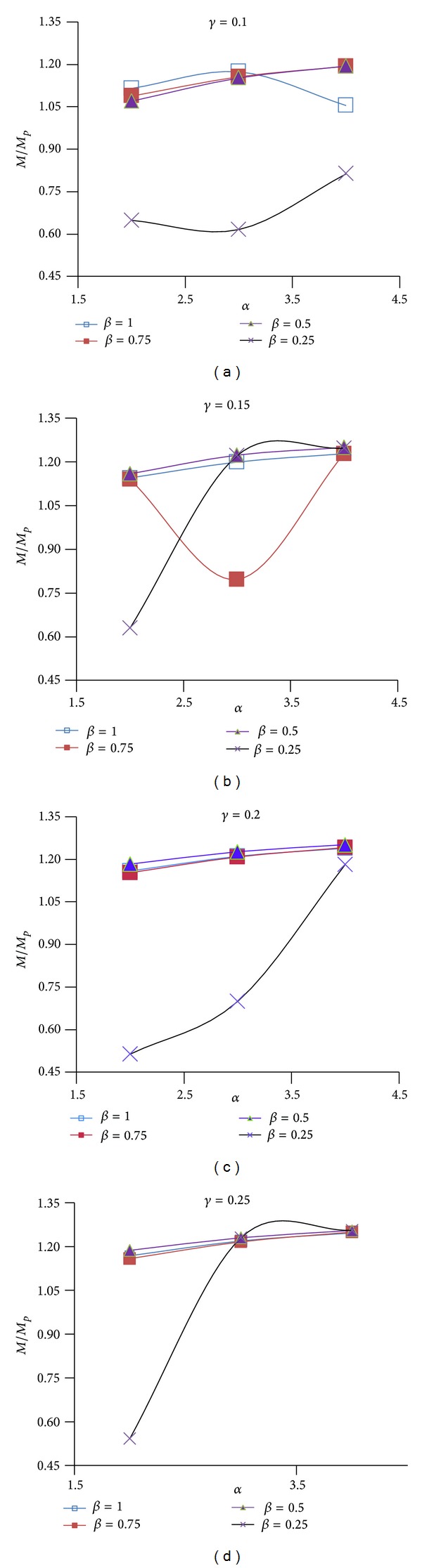
*M*/*M*_*P*_ versus *α* for different values of *β* and *γ* for SAC3.

Figures 12, 13, and 14 show the effect of design parameters *α*, *β*, and *γ* on the strength of the modified specimens SAC7, SAC5, and SAC3. Connection strength was evaluated by using the *M*/*M*_*P*_ ratio which is the ratio of the applied moment measured at the column face level at the failure time to the full beam plastic moment capacity at the column face level. This comparison was done for monotonic loading. 

As shown in Figures 12 to 14 for most of the modified specimens (those of parameter *β* equal to 1, 0.75, and 0.5 and any value of parameter *γ*) the increase in parameter *α* caused a gradual increase in the connection strength. This was due to the decrease in the depth of the first pair of voids, and consequently the increase in the depth of the T-sections (parameter *b*_1_) remained at the top and bottom of the first rectangular voids which finally resulted in the enhancement of the flexural and torsional stiffness of the T-sections. For specimens of *β* equal to 0.25, increase in parameter *α* caused a significant increase in the connection strength. It should be note that for these specimens the second pair of horizontal voids was located at the nearest distance from the top surface of the beam flange. In other words in these specimens the T-sections remained at the top and the bottom of the second pair of rectangular voids had the smallest depth and largest slenderness ratio. As a result, for these specimens, increase in parameter *α* indirectly decreased the slenderness ratio of the second T-sections and caused the highest increase in the connection strength. However, specimens of *β* equal to 0.25 had the smallest ductility in compare to the other specimens. 

As it is clear from the Figures 12 to 14, the decrease in parameter *β* (from 1 to 0.5) had a very small effect on the connection strength degradation. For most of these specimens, the value of *M*/*M*_*P*_ ratio was greater than 1.05. However, the excessive decrease in this parameter (i.e., *β* = 0.25) caused a remarkable reduction in the connection strength which was due to the excessive increase in the slenderness ratio of the T-sections remained at the top and the bottom of the second pair of the rectangular voids. As these figures show, by increasing parameter *γ* and consequently increasing the clear distance between the voids, the connection strength slightly increased. However, as mentioned above it caused a reduction in the connection strength.

### 4.3. Summary of Results

Based on the ANSI/AISC 341-10 [30], beam-to-column connections used in the seismic force resisting system (SFRS) shall satisfy the following requirements.

1. The connection should be capable of accommodating a story drift angle of at least 0.04 rad.
2. The measured flexural resistance of the connection, determined at the column face, should be equal to at least 0.80*M*_*P*_ of the connected beam at a story drift angle of 0.04 rad.

Based on the discussion presented in the previous section and with respect to the requirements mentioned in the above paragraph, from the strength and ductility point of views, for all modified SAC specimens, the highest connection performance might be achieved for *α* = 2, *β* = 0.75, and *γ* = 0.1. The specimens under consideration for this study easily achieved the minimum required strength. The maximum ductility achieved for these specimens was 5.0, 4.08, and 4.08 percent radian for beam depths of 912 mm, 750 mm, and 600 mm, respectively which are all greater than the minimum required ductility. 

Despite of the increase in the ductility of the specimens SAC5 and SAC3 (the ductility of nonmodified specimens SAC5 and SAC3 are 0.02 and 0.03 radian, resp. [18]), the results indicate that the proposed BEC might be much more effective for deep beams rather than shallow beams. The reason can be as follows. The usage of the longitudinal voids is the key parameter for the enhancement of connection ductility for the BECs presented in [18] and in this study. In this study, the length of voids was considered as 1.25 times the beam overall depth. Hence, for deeper beams the voids were longer and dissipated more seismic energy when compared to the shallower beams where the voids are shorter. This might be the reason for deeper beams achieving higher ductility than the shallower beams of the proposed BEC. 

## 5. Generalization of the Design Procedure 

The modification procedure presented in the previous section is easy for application. However, a few key parameters, such as gravity effect, length of the beam and moment gradient of the beam, were neglected. This section considers the use of these parameters to generalize the design procedure so that the proposed modifications can be applicable to other sections. This design procedure is based on the one presented in FEMA350 [31] and is similar to the one presented by Engelhardt et al. [32] for RBS and welded haunch connections.

The design method is based on the limiting moment, *M*_pd_ (see (5)), and the associated shear force, *V*_pd_ (see (6)), at critical plastic section, which is the starting point of the first pair of voids. The critical plastic section is denoted in Figure 3 by parameter *S*_*C*_ and can be obtained by using (7). Consider the following:

(5) $$\begin{array}{c} M_{\text{pd}}=C_{\text{pr}}\times Z_{\text{RBWS}}\times F_{\text{ye}}, \end{array}$$

(6) $$\begin{array}{c} V_{\text{pd}}=\frac{2M_{\text{pd}}}{L^{\prime }}+\frac{wL^{\prime }}{2}, \end{array}$$

(7) $$\begin{array}{c} S_{C}=D-\frac{L_{v1}}{2}+r_{v}, \end{array}$$

(8) $$\begin{array}{c} Z_{\text{RBWS}}=Z_{b}-D_{v1}\times t_{w}\times \left(a_{1}+D_{v1}\right). \end{array}$$

In these equations *F*_ye_ is the expected yield stress of material, *Z*_RBWS_ is plastic section modulus at RBW area (see (8)), *w* is the factored gravity loads on the beam, and parameter *L*′ is shown in Figure 3. The moment at the column face, *M*_*f*_, is

(9) $$\begin{array}{c} M_{f}=M_{\text{pd}}+V_{\text{pd}}\times S_{C}. \end{array}$$

By substituting (5) and (6) into (9) and normalizing both sides with respect to the full section plastic moment of beam (*Z*_*b*_ · *F*_ye_), the maximum normalized moment at column face, *η*_*t*_, will be

(10) $$\begin{array}{c} \eta_{t}=\eta_{g}+\eta_{e}=\frac{w\times L^{\prime }\times S_{C}}{2Z_{b}\times F_{\text{ye}}}+C_{\text{pr}}\times \frac{Z_{\text{RBWS}}}{Z_{b}}\left(1+\frac{2S_{C}}{L^{\prime }}\right). \end{array}$$

The first term, *η*_*g*_, considers the effect of gravity loads and the second term, *η*_*e*_, considers the effect of seismic loads. For simplicity, the influence of the portion of gravity load within the length *S*_*C*_ was neglected. To enhance the ductility of a post-Northridge connection, the configuration of voids (or the values of parameters *α*, *β*, and *γ*) must be chosen as such to keep the value of *η*_*t*_ in an appropriate range to avoid beam flange fracture at both RBW area and WAH region before achieving adequate connection ductility, 4% total rotation at column web center.

For any modified connection *η*_*t*_ can be determined using experimental and analytical results. For example, *η*_*t*_ is 1.05 [32] and 1.15 [31] for RBS connections of bottom flange cut and both top and bottom beam flange cut, respectively. However, in the study done by Hedayat and Celikag [18] for RBW connections with single rectangular voids, depending on the beam overall depth and the connection type, a range of appropriate *η*_*t*_ values (between 1.05 and 1.14) was proposed. The normalized moment, developed at the column face, *η*_*t*_ = *M*/*M*_*P*_, for all modified specimens, was graphically shown in Figures 12 through 14. Appropriate values of parameter *η*_*t*_ can be determined by comparing its values (Figures 12 to 14) with the connection ductility, *θ*_cwc_ (Figures 9 to 11). Despite being difficult to select, based on the data presented in Figures 9 to 14, a value between 0.95 and 1.02 might be the best value of parameter *η*_*t*_. However, according to the finite element results, a value closer to the lower bound might be more appropriate for deeper beams (beam depth ≥ 750 mm), while a value closer to the upper bound might be more suitable for shallower beams.

*C*_pr_ in (5) is a factor to account for the peak connection strength, including strain hardening, local restraint, and additional reinforcement. In FEMA350 [31], the *C*_pr_ factor is given by equation (*f*_*y*_ + *f*_*u*_)/2*f*_*y*_, where *f*_*y*_ and *f*_*u*_ are the specified minimum yield and tensile stress of material, respectively. FEMA350 [31] proposes the use of value 1.2 for any case of modified connections except where otherwise noted in the individual connection design procedure. This factor is the ratio of the measured moment at the starting point of the first pair of voids (i.e., at the critical plastic section) at the connection failure time to the beam plastic moment capacity at this location. For the proposed BEC, this factor is a function of the configuration and the size of voids. Therefore, in this study the nonlinear model given in (11) was used to estimate the *C*_pr_ factor, based on the design parameters *α*, *β*, and *γ* and beam flange and web slenderness ratios. In this equation *b*_*f*_, *t*_*f*_, and *t*_*w*_ are the beam flange width and thickness and the beam web thickness, respectively. Constant *C*_1_ and exponents *C*_2_ to *C*_6_ were determined using regression analyses and summarized in Table 3. The last column of Table 3 gives the observed average error. This error is the mean square error which emphasizes the effect of large errors (∑_1_^*n*^(real  *C*_pr_ − estimated  *C*_pr_)^2^, *n* is total number of data). Consider

(11) $$\begin{array}{c} C_{\text{pr}}=C_{1}\times \alpha_{}^{C_{2}}\times \beta_{}^{C_{3}}\times \gamma^{C_{4}}\times {\left[\frac{D-2t_{f}}{t_{w}}\right]}^{C_{5}}\times {\left(\frac{b_{f}}{t_{f}}\right)}^{C_{6}}. \end{array}$$

After finalizing the geometry of proposed BEC, the connection ductility can also be estimated using (12). Constant *C*_1_ and exponents *C*_2_ to *C*_7_ were determined using regression analyses and summarized in Table 3. The last column of Table 3 gives the average of mean square error observed for all SAC specimens. Consider

(12) θCWC=C1×αC2×βC3×γC4×[D−2tftw]C5×(bftf)C6×(LbD)C7.

Note that in order to use (11) and (12), all design principles presented in Section 4 should be considered (i.e., *L*_*v*1_ = *L*_*v*2_ = 1.25*D*; *D*_*v*1_ = *D*_*v*2_; *a*_1_ = 5.29 × *Z*_*b*_/(*L*_*b*_ · *t*_*w*_); *a*_2_ ≥ *a*_1_, and the distance from the center of the first pair of voids to the column face is equal to the beam overall depth, *D*). 

## 6. Cyclic Loading Effects

**Figure 15:**
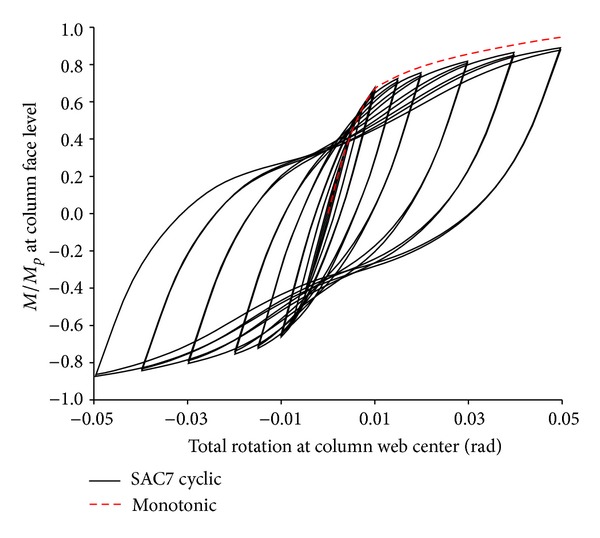
Normalized moment rotation curve of specimen SAC7 for *α* = 2, *β* = 0.75, and *γ* = 0.1.

**Figure 16:**
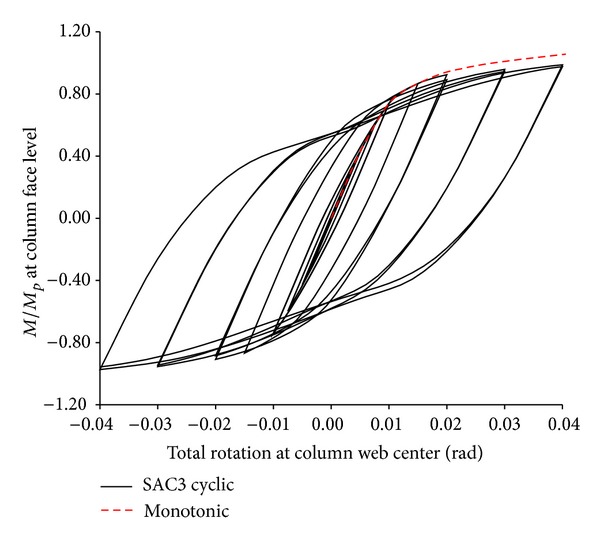
Normalized moment rotation curve of specimen SAC3 for *α* = 2, *β* = 0.75, and *γ* = 0.1.

Under cyclic loading, connection strength is generally lower than the one obtained under monotonic loading. It is due to beam flange and web local buckling. In this study, all specimens of the adequate strength and ductility (i.e., those specimens of the optimum values of parameters *α*, *β*, and *γ*) were reanalyzed under cyclic loading to determine the amount of strength degradation. For instance, in Figures 15 and 16 the moment-rotation curves of the modified specimen SAC7 and SAC3 for *α*, *β*, and *γ* equal to 2, 0.75, and 0.1, respectively are compared for both monotonic and cyclic loading. It can be seen from the figures that there was no remarkable difference between the connection strength obtained from the two loading types. There was only a small amount of reduction in the connection strength which was due to the local buckling of the beam web at the RBW area. For both modified specimens SAC7 and SAC3, the connection strength is greater than the minimum required strength, *M*/*M*_*P*_ = 0.8.

Figure 15 also shows a pinching in the hysteresis curve of specimen SAC7 which was due to the beam flange/web buckling at the RBW area. By decreasing the beam overall depth, for shallower beam specimen SAC3, the amount of pinching significantly reduced. The hysteresis curve was more stable with an insignificant amount of pinching, and this was also due to the increase in the parameters *α*, *β*, and *γ*. 

Similar behavior was also observed for modified specimen SAC5. Hence, it might be concluded that the modified SAC specimens have adequate strength to be used in seismic regions. Therefore, as it was assumed in the analytical studies performed under monotonic loadings by Ricles et al. [33] and El-Tawil et al. [34], the conclusions drawn from the monotonic loading might be qualitatively applicable to cyclic conditions.

## 7. Conclusion

The aim of this study was to find practical and effective ways to enhance the ductility and strength of post-Northridge connections so that they are better applicable for new and existing buildings. For this purpose multilongitudinal voids horizontally opened in the beam web where the distance of the centerline of the first pair of voids from the face of the column was equal to the beam depth. All voids had the same length (1.25 times the beam overall depth) and the same depth. Design parameters *α*, *β*, and *γ* were defined to change the geometrical location of the voids. A parametric study was carried out with respect to these parameters to find the optimum location of voids to achieve the highest connection strength and ductility. This finally led to the modeling of the 144 post-Northridge specimens of different beam overall depths. Analytical results showed that the presence of the second pair of voids was efficient in uniformly distributing the plastic equivalent strains along the beam length and, therefore, significantly reducing the plastic equivalent strain concentration at the column face level, weld access hole region, and at the beam flanges at the void areas. It finally led to the achievement of the adequate strength and ductility for the specimens of the proposed BEC. Results also showed that the location and size of voids can influence the performance of the modified connections. The effect of the configuration of voids was investigated using design parameters *α*, *β*, and *γ*. Results indicated that the highest connection strength and ductility can be achieved for *α*, *β*, and *γ* equal to 2, 0.75, and 0.1 respectively. These specimens achieved the minimum required strength. From the ductility point of view, however, the proposed method caused a remarkable increase in the ductility of all connections when compared to the single pair voids [18]. They all achieved the minimum required ductility. On the other hand, the efficiency was better for the deeper beams (overall depth greater than 750 mm) where the deep beam specimens SAC7 (overall depth equal to 912 mm) achieved a remarkable five percent total rotation.

In order to generalize the design procedure to be applicable to any other beam section, estimate the best configuration of voids, and also to consider other design parameters, (beam length, beam moment gradient and beam gravity loads) (11) and (12) were proposed. Finally, the best location of voids was controlled by using the parameter *η*_*t*_. It is expected that any modified specimen (even in the case of a shallow beam) with appropriate value of parameter *η*_*t*_ (0.95≤, *η*_*t*_ ≤ 1.02) achieves both adequate connection's strength and ductility simultaneously.

## Figures and Tables

**Table 1:** Geometric parameters of SAC specimens.

Specimen	Shear tab (mm)	No. of A325 SC bolts (mm)	Continuity plate (mm)	Weld type and size (mm)	
				Beam flange	shear tab
SAC3	457 × 127 × 9.5	6Φ22	305 × 127 × 16	CJP, root opening = 9 mm, bevel angle = 30° and E70TG-K2	Fillet, 8 mm, E70T-7
SAC5	610 × 127 × 12.7	8Φ25	305 × 127 × 19		
SAC7	762 × 127 × 15.9	10Φ25	305 × 152 × 25.4		

**Table 2:** Material properties of the SAC specimens (MPa).

Specimen *F*_*y*_/*F*_*u*_	Beam		Column		Shear tab	Continuity plate
	Flange	Web	Flange	Web		
SAC3	315.2/468.1	340.9/480.6	319.4/469.4	345.8/475.0	323.6/490.3	358.3/509.7
SAC5	355.5/484.7	382.6/497.2	360.4/511.1	356.2/500.3	288.9/446.5	302.1/444.4
SAC7	290.3/441.7	327.1/447.2	335.4/490.3	306.9/475.7	358.3/509.7	310.4/475.7

**Table 3:** Variables *C*_1_ to *C*_7_ to predict *C*_pr_ and *θ*_CWC_.

Equation	*C*_1_	*C*_2_	*C*_3_	*C*_4_	*C*_5_	*C*_6_	*C*_7_	Err
Equation (11) to predict *C*_pr_	1.8231	0.1718	0.1214	0.1184	−0.7828	0.9854	—	0.018040
Equation (12) to predict *θ*_CWC_	2.4678	−0.1583	0.1066	−0.1897	1.1905	−1.4761	−0.2964	0.004809
